# Cell-based fish production case study for developing a food safety plan

**DOI:** 10.1016/j.heliyon.2024.e33509

**Published:** 2024-06-22

**Authors:** Reza Ovissipour, Xu Yang, Yadira Tejeda Saldana, David L. Kaplan, Nitin Nitin, Alex Shirazi, Bill Chirdon, Wendy White, Barbara Rasco

**Affiliations:** a Department of Food Science and Technology, Texas A&M University, College Station, TX, 77843, USA; b Department of Nutrition and Food Science, California State Polytechnic University, Pomona, CA, 91768, USA; c New Harvest Canada, Cochrane, T4C 0V2, AB, Canada; d Department of Biomedical Engineering, Tufts University, Medford, MA, 02155, USA; e Department of Food Science and Technology, University of California-Davis, Davis, CA, 95616, USA; f SVCMS, LLC, San Francisco, California, USA; g Kunzler & Company Inc., Lancaster, PA, 17604, USA; h Georgia Manufacturing Extension Partnership (GaMEP), Georgia Tech, Atlanta, Georgia, USA; i College of Agriculture and Natural Resources, University of Wyoming, Laramie, WY, 82071, USA

**Keywords:** Cell-based fish, Cultivated seafood, Cultured seafood, Food safety plan, HACCP, Cell culture

## Abstract

Given the expanding global population and finite resources, it is imperative to explore alternative technologies for food production. These technologies play a crucial role in ensuring the provision of safe, nutritious, and sustainable food options to meet the growing demand. Cellular agriculture plays an important in developing an alternative method for developing food products. While, cellular agriculture is emerging rapidly, food safety aspects and regulatory frameworks stayed behind. Despite developing several regulatory framework papers on cellular agriculture, there is no systematic approach for developing a comprehensive food safety plan (FSP), particularly for cultivated seafood. Thus, the overall goal of this article is to develop a FSP for cultivated seafood. The main differences between the food safety plan for cultivated seafood and the conventional seafood industries were the number of allergens in cultivated seafood products, including soy, wheat, and fish cells, compared to only fish for the conventional seafood industry. In addition, there are several hazards associated with mycoplasma in cultivated seafood, which should be considered. This guidance intends to help regulatory agencies, food safety experts, startup companies, and the cultivated seafood industry by providing a valuable platform to develop regulations, guidance, and food safety plans applicable to most cultivated seafood companies. This article will also help the industry to identify the hazards in their processing line and develop preventive controls, and as a comprehensive food safety plan, it could be easily adapted for other cultivated seafood products. This guidance applied systematic approaches to developing food safety plans using cell culture, pharmaceuticals, fermentation, seafood, meat, and aquaponics safety plans, collaborating with experts with different backgrounds, and working closely with the conventional and cultivated meat and seafood industries.

**Table:** Abbreviations and acronyms

ATP	Adenosine Triphosphate
CCP	Critical Control Points
CFR	Code of Federal Regulations
CITES	Convention on International Trade in Endangered Species (CITES)
DNA	Deoxyribonucleic Acid
FAO	Food and Agriculture Organization of the United Nations
FDA	Food and Drug Administration
FSMA	Food Safety Modernization Act
GFI	Good Food Institute
GMPs	Good Manufacturing Practices
CGMPs	Current Good Manufacturing Practices
GRAS	Generally Recognized as Safe
HACCP	Hazard Analysis and Critical Control Points
IACUC	Institutional Animal Care and Use Committee
N/A	Not Applicable
OTR	Oxygen Transmission Rate
QC	Quality Control
RLU	Relative Light Unit
SOP	Standard Operating Procedure
SSOP	Sanitation Standard Operating Procedures
WHO	World Health Organization
US	United States
USA	United States of America

## 1 Introduction

The demand for meat and animal-based products is increasing dramatically as the world population is predicted to grow to 10 billion by 2050, as estimated by the FAO. To address this demand, the total food and meat production must increase by 70 % and 100 %, respectively [1]. However, the conventional meat production system is facing issues in meeting this demand due to the limited resiliency, inefficacy in water and land use, as well as increased emission of greenhouse gases.

Cell-based food production may offer possible solutions to address immediate societal problems by developing new sustainable agri-food systems to feed a rapidly growing global population with nutritious and safe food while reducing the environmental impact and resource usage (78–96 % less greenhouse gas emissions, 99 % less land, and 82–96 % less water use) [2,3]. Nonetheless, one of the important prerequisites in the cell-based food production is food safety assurance.

According to the WHO report, 31 foodborne agents, including bacteria, viruses, parasites, toxins, and chemicals, are responsible for more than 600 million cases of foodborne illnesses and 420,000 deaths annually [4]. The burden of foodborne diseases falls on groups in vulnerable situations and especially on children under 5, with the highest burden in low- and middle-income countries [4]. Thus, due to the importance of food safety and the novelty of cell-based food production systems and their food supply chain, utilizing the existing framework and modifying them to ensure the safety of cell-based food is necessary. In order to determine the food safety concerns in any food processing system, a case study on developing a specific food safety plan can be beneficial and serve as a basic document that can be easily modified by users for other food products.

The cell-based meat and seafood industry is growing rapidly worldwide, while regulations are slowly developing. In Europe, this industry falls under the Novel Food Regulation or Genetically Modified Organisms if genetic modification is a part of the process. In the US, FDA and USDA agreed to create a joint regulatory approach in 2018, followed by establishing a joint agreement in 2019 to address the cultivated meat and seafood regulations.

FDA oversees cell collection, cell banks, cell growth, and differentiation, while USDA/FSIS will look after the harvest stage onwards. Codex Alimentarius also recently initiated programs on developing HACCP and GMP for cultivated meat and seafood. At this moment, many cultivated meat and seafood companies in the US prepare their food safety plan and standards based on both FDA and USDA regulations.

Cultivated meat and seafood need to comply with the preventive control rules established by the FSMA. According to FDA, “Generally, domestic and foreign food facilities that are required to register with section 415 of the Food, Drug, & Cosmetic Act must comply with the requirements for risk-based preventive controls mandated by the FDA FSMA as well as the modernized CGMPs of this rule (unless an exemption applies)”. It is important to note that the applicability of the CGMPs is not dependent upon whether a facility is required to register. This rule, which became final in September 2015, requires food facilities to have a food safety plan in place that includes an analysis of hazards and risk-based preventive controls to minimize or prevent the identified hazards. In addition, all facilities subject to Subpart C must have a Preventive Controls Qualified Individual available to perform or oversee the responsibilities. A Food Safety Plan consists of the primary documents, including products description, production flow chart, process description, hazard analysis, preventive controls (allergen preventive controls, preventive sanitation controls, preventive temperature controls), verifications (facility sanitation verification), and implementation, in a preventive controls food safety system that provides a systematic approach to the identification of food safety hazards that must be controlled to prevent or minimize the likelihood of foodborne illness or injury [[5], [6], [7], [8], [9]]. Traditionally, the conventional seafood industry is regulated by the FDA, except for catfish, which and meat products are regulated by USDA [10]. Over the past three years, numerous companies have engaged with regulatory agencies across various countries to secure approvals for the production and sale of safe cell-based meat products. Most of these companies such as Upside Foods, Good Meat, Just, Aleph Farms, Vows, and Mosa meat, have been mainly focusing on cell-based meat from terrestrial animal [11,12]. The novelty of cell-based food production processes and products raises significant concerns regarding food safety and food safety regulations. Additionally, national competent authorities must grapple with various socioeconomic considerations related to these products. These include consumer preferences, acceptance, ethical considerations, production costs, trade matters, and market pricing.

Given the need for clear product labeling and potentially special authorization processes, regulatory frameworks may require adjustments or even new implementations. This is crucial as cell-based food products may enter jurisdictions or cross borders at any time. Therefore, ensuring the safety and proper regulation of these novel food items is of paramount importance.

During the last five years, several articles were published on regulatory frameworks of the cell-based meat. For example, researchers evaluated the food safety considerations and research priorities for cell-based meat [13], cell-based meat safety research priorities with an emphasis on regulatory and governmental perspectives [14], and policy frameworks, and regulation [11]. Food safety aspects of the cell-based meat have also been reviewed and developed [15,16].

One of the major component of the regulatory framework, is distinguishing the hazards during the production, and developing preventive controls based on that. The foundation of the meat and poultry industry's proactive food safety strategy lies in a system known as HACCP. HACCP originated in the 1960s when Pillsbury developed it for the food industry, aiming to ensure the safety of food for the burgeoning space program. Over the past half-century, this system has been widely embraced by numerous food processors and is regarded as the ‘gold standard’ for managing food safety processes.

Using HACCP, meat and poultry companies conduct thorough analyses of their production processes for each product they manufacture. They identify CCPs - these are specific stages within the production process where potential biological, physical, and/or chemical hazards can be effectively managed. A CCP might involve ensuring that meat is properly cooled to control bacterial growth or that ready-to-eat products like deli meats are cooked sufficiently to eliminate bacteria. Once these CCPs are pinpointed, companies implement, monitor, and document their control measures. This documentation is crucial for ensuring that their food safety system operates effectively. While, HACCP is a useful tool to minimal the risks, a Food Safety Plan (FSP) serves as the cornerstone of a preventive controls food safety system. It encompasses key documents that form a systematic strategy for recognizing food safety hazards that require management to reduce the risk of foodborne illnesses or injuries. This plan comprises a comprehensive set of written materials outlining the procedures and practices implemented to guarantee the safety of food throughout its various stages, including manufacturing, processing, packaging, and storage. The goal is to reduce the chance of someone getting sick from eating contaminated food. According to the U.S. Food Safety authorities (USDA and FDA), FSP must be prepared by Preventive Controls Qualified Individuals and each food processing companies must have their own FSP. The differences between HACCP and FSP are listed in Table 1.

**Table 1:** Differences between HACCP and FSP.

Parameter	HACCP	FSP
Hazard analysis	B, C, P	C: Radiological, economically motivated fraud
Parameters & Values	CCPs limits	Parameters and m/m of the CCPs
Monitoring	Required for CCPs	Required as appropriate for PC
Corrective actions and Corrections	Corrective actions	Corrective actions or corrections as appropriate
Verification	For process controls	Verification as appropriate for all preventive controls; validation for process controls; supplier verification is required when the supplier controls a hazard
Records	For process controls	As appropriate for all preventive controls
Recall plan	Not required in the plan	Required when a hazard requiring preventive control is identified

**Fig. 1:**
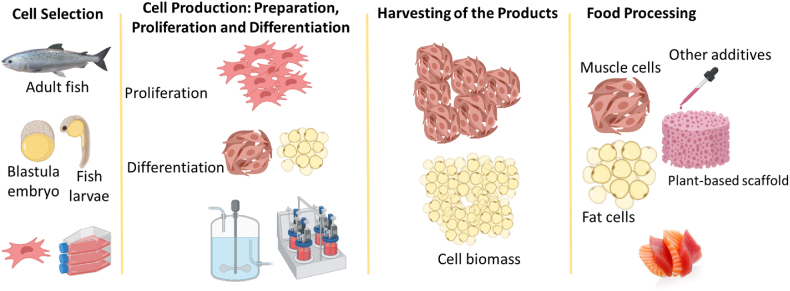
Example of a process for cell-based fish production.

This document aims to provide a case study on developing a food safety plan for a cell-based fish product. In order to develop a FSP, we developed a conceptual case, and did not use any data from an existing company. The first step of developing a food safety plan is to fully understand the entire production process, from cell sourcing to food processing (Fig. 1), and identify potential hazards so that respective risk assessments can be conducted. As food safety can only be achieved by all the players in the food chain, this case study was developed by a team of biomedical engineers, food safety engineers, food safety specialists, and regulatory experts. In addition, it was also beneficial to obtain insights from the perspectives of tissue engineering and cell culture manufacturing practices (for human studies), the pharmaceutical industry, fermentation, brewery industry, and aquaponics industry [[17], [18], [19]]. Food safety risks associated with cell-based ground beef has already been developed and discussed (GFI). Since, seafood products poses more risks in terms of food safety, and no FSP has been developed for the cell-based seafood, this study will focus on cell-based Atlantic salmon. The case study was developed in the US thus, relevant regulatory frameworks exiting in the US have been mainly considered for compliance issues.

## 2 Cell-based fish product scenario

### 2.1 Background

This is a food safety plan for a cell-based salmon production illustrating how technical food safety matters and regulatory compliance issues can be considered in developing a plan. Regulatory requirements vary among different countries and jurisdictions. Therefore in this case study, relevant regulations in the United States of America (USA) were referred to develop a scenario. In particular, references were made to the regulations in 21 Code of Federal Regulation (CFR) Part 117 & 123 associated with the Food Safety Modernization Act (FSMA), PL 111–353 that applies in the United States (US) Conditions and specifications used (*e.g*., validation information) for illustrative purposes only [9]. As the real-life pre-market authorization has not been made in the USA, the compliance conditions described in the case study may not completely represent actual process conditions.

### 2.2 Hypothetical company overview

Cell-Based Salmon Company is a cell-based food production company producing cell biomass and fish portions (whole cut). The company produces 1000 kg of cell-based fish per day and sells fish cell biomass to local restaurants and other cell-based food manufacturers; and fish portions to local restaurants, grocery stores, and consumers. The products are shipped to local distributors and retailers in refrigerated or frozen conditions.

### 2.3 Product descriptions

This section was prepared based on the standard food safety plan documents in the US, which started with product descriptions. In this section, companies provide details about the specific products they produce. Depending on the number of products, there will be individual tables representing each product (Table 2). Food safety plans are developed by experts, quality assurance managers, food safety directors, or third-parties. In our case study, a mock company (Cell-based Salmon) was used to represent the case study. Since this case study is related to a mock company with two products (fish cell biomass and fish portion), there needs to be two different tables representing each product, while, to save the space, both products are combited in one table.

**Table 2:** Product description for cell biomass.

Product Description	Atlantic salmon (*Salmo salar*) – Fish Cells
Cell-line developer	
Product developer	
Producer	
Date of submission	
Product name	Atlantic salmon (*Salmo salar*) – cell biomass/fish portion
Ingredients	*Cell Culture Media*: Green algae (Chlorella, Spirulina) extracts; soy hydrolysates (an allergen); yeast hydrolysates; mushroom hydrolysates; pea hydrolysates; corn hydrolysates; sorghum concentrate and hydrolysates; wheat hydrolysates (an allergen), glucose, glutamine; Algae-based oil; fungi-based oil; Vitamin C; B Vitamins; Insulin; Transferrin; Albumin (source, if egg, then an allergen). *Fish Muscle Cells*: Atlantic salmon (*Salmo salar*) cell biomass *Fish Fillet*: Atlantic Salmon (*Salmo salar*) Portions: Mince, skinless, and boneless fillet, individually vacuum packed. Other Ingredients: soy (an allergen), pea, wheat (an allergen), microbial-based collagen, yeast extract, algae astaxanthin (from *Haematococcus pluvialis*), plant-based nano-fibers (Cellulose), alginate, carrageenan, xanthan, curdlan.
Packing information	Weight (25 lb per box) of 2–4 oz vacuum packaged (10 K OTR film), or non-vacuum package weight-controlled portions. Keep refrigerated at 38 °F or less by commercial refrigeration or with gel ice. Boxes are sealed with a tape having the company logo as a tamper-evident feature and have printed lot code, species, weight, date of harvest, and company address information.
Intended use	For further processing by manufacturers or as an ingredient for restaurants and consumers.
Intended users	The general public, to be cooked prior to consumption.
Shelf life	5–7 days at (≤38 °F/3 °C)
Allergens	Fish (Atlantic Salmon), soy, wheat, albumin if it is from egg
Suggested labeling instructions	Keep refrigerated; Contains fish (Atlantic Salmon); to be cooked prior to consumption; Contains allergens including fish, soy, wheat; Keep frozen, and Remove from film before thawing for vacuumed products.
Other items that can appear on the labeling, as applicable	Lot number, best use-by date, intact container sealing tape as a tamper-evident feature. Species listed (allergen warning for Salmon, soy, wheat, albumin if from the egg).
Storage and distribution	Keep refrigerated (≤38 °F/3 °C)

### 2.4 Production flow diagram

**Fig. 2:**
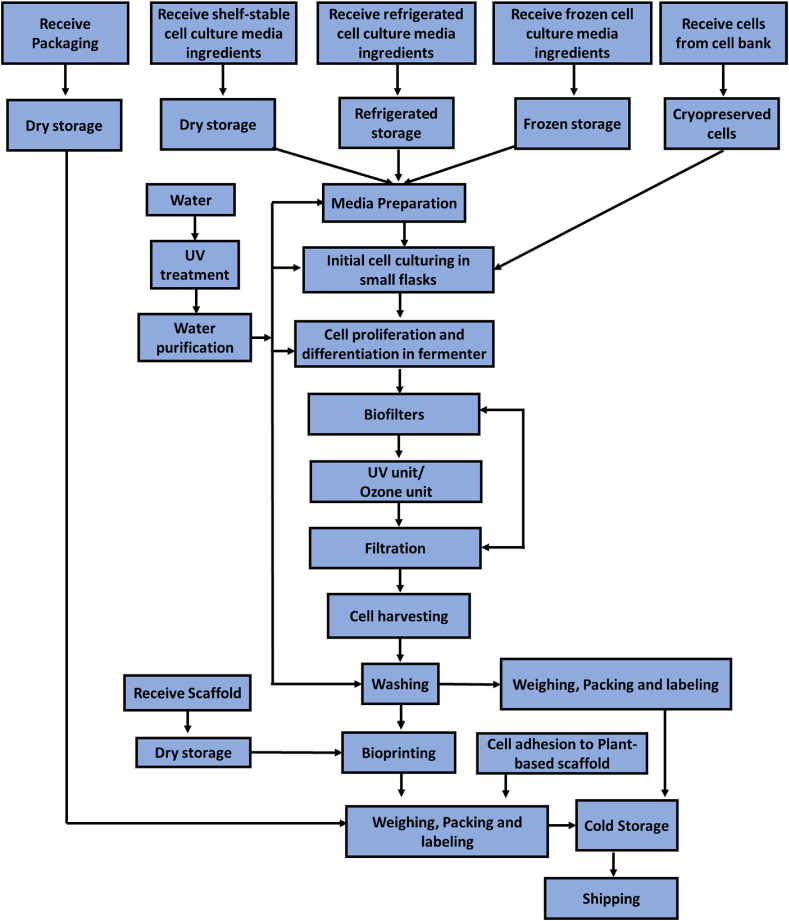
Cell-based fish cell biomass and fish portions flow chart.

Developing flow diagram of the production chain for each product could be beneficial for identifying the hazards and critical points to develop preventive controls measurements and improve food safety. Companies develop flow diagrams for each product individually. For example, in this document, there are two products, including fish cell biomass and fish portions (Fig. 2).

### 2.5 Process description – cell sourcing and receiving materials

For process description which is based on the flow diagram (Fig. 2), every step of the process must be described in details. We have provided the detailed description for all the process steps presented in Fig. 2.

#### 2.5.1 Receiving packaging materials

Packaging materials are inspected for signs of odor, damage, or contamination. Labels will be checked to ensure all the details are correct and there is no sign of contamination. A certificate of analysis indicating that the materials are safe as food contact will be provided by the supplier. Packaging materials should be stored covered in a segregated area away from the rest of the plant, and any stored allergenic materials or chemicals. Temperatures should not exceed 110 °F/44 °C. Different packaging materials will be used, for example, FDA-approved 10,000 oxygen transmission rate (OTR) will be used for fish cell and fish portion packaging, and paper-based boxes will be used for secondary packaging.

#### 2.5.2 Receiving cells (*i.e*., from the cell bank)

Cells will be provided by an independent cell bank or our company's cell bank. Cells will be from a healthy animal confirmed to be devoid of potential toxicants (*e.g*. mercury, ciguatera toxin, and harmful algae toxins if the cells are from marine organisms).

Cell line banks can always store the original cell lines for further analysis if necessary. In the case of induced pluripotent stem cell lines (iPSC), based on good cell culture practices, it is also good to cryopreserve the original cells used. The vendors can also provide a certificate from the Convention on International Trade in Endangered Species (CITES) indicating that the animal origin is not listed under CITES.

Healthy cryopreserved immortalized Atlantic salmon cell lines will be thawed upon arrival in the production facility at temperatures not exceeding 38 °F/4 °C for culture in sterile, food-grade T-25 and T-75 flasks. The quality of the cells in terms of microbial contamination, mycoplasma contamination, doubling time, morphology, and survival will be evaluated prior to assigning a lot number. Any flasks of propagated cells that are contaminated with bacteria or mycoplasma will be destroyed with destruction documented. A health certificate and species authentication (DNA barcode) will be requested if the cells are received from a vendor and supplier.

**Note**: Transmissible spongiform encephalopathies (TSEs) or prion disease are potential hazards *only* when culturing bovine cells and are not a hazard concern for aquatic animal species.

**Note**: Selecting the cell line for the cell-based food is the first step in the process. Several criteria have been developed for selecting the proper species for cell line development [20]. The cell lines should be sourced from a certified provider, such as established cell banks, universities, research laboratories, commercial providers, and culture collections. If documentation and certificates regarding the origin and quality of the cell lines are not provided, then the facility must test the cell lines. Documentation of the absence of contamination by major classes of biological contamination (*e.g*., mycoplasma, bacteria, fungi, and viruses), genetic identity/consistency/traceability, and stability of desired functionality should also be available (Table 3).

**Table 3:** Helpful information to document regarding the cell lines.

Parameters	Isolated organs and tissues of animal origin (*e.g.* Atlantic salmon muscle tissue)
Ethical and safety	Animal welfare
Species/strain	e.g. Atlantic salmon
Source	Wild/Aquacultured
Gender (if applicable)	Female/male
Age (if applicable)	Blastula/larvae/adult
Any chemical pretreatment	Drugs/Antibiotics
Organ/tissue of origin	Muscle/fat tissue
Isolation technique	Biopsy
Date of isolation	
Supplier	
Pathogen contamination	*Listeria* sp., *Aeromonas* sp.,
Mycoplasma contamination	
Recommended temperature	28 °C
Recommended medium	L15 + Peptone
Images	
Permits and restrictions	
Handling procedure attached	
Subculturing procedure attached	
Cell morphology	Muscle cells
Growth Properties	Fast
Histopathology	
Phenotype	
Differentiation	Positive
Growth and doubling time	2 days
Initial passage number	10 times

Biological materials, including cell lines and tissues may fall into the “dangerous goods” for shipping purposes. In this case, they must comply with national regulations and/or international platforms such as the International Air Transport Association (IATA) transport regulations and/or the Dangerous Goods Regulations (DGR). Diagnostic specimens of human or animal material, including (but not limited to) blood and its components, tissue, tissue fluids, or body parts, are generally classified as Biological Substance, Category B (UN3373) for air transport. Since the cell-based food industries may depend on transporting cells and tissues worldwide, it is critical to keep the samples as healthy as possible during transportation. Cells and tissues may need to be shipped in a particular temperature-controlled environment, such as a mini-cell culture incubator, which has limited space (2–3 plates or flasks) and requires adequate sealing of the plates to avoid leakage. These may not always be an option or available, so for short trips (*e.g*., arriving within one working day), it may be possible to ship cell lines in culture medium-filled flasks.

For cells shipped on dry ice or liquid nitrogen using specific containers, the cell condition should be examined and documented on arrival at the destination.

##### 2.5.2.1 **Note:***In-house cell line development*

Many cell-based meat companies develop cell lines from different species, including avian, mammalian, and aquatic animal species. If the facility has access to animal sources, the providers, including slaughterhouses (health certificate about animal status is necessary), research facilities, aquaculture farmers, and fishermen, should provide IACUC approved protocols. If the facility has an animal husbandry system, the facility and those working with animals should be approved by IACUC.

The number of animals per space (density), animal welfare, age of the animals, and their disease history should be recorded and monitored since these parameters impact the cell lines.

Cell line development has three broad categories, including isolated organs or tissue, primary and early passage cultures, and cell lines (finite, continuous, and stem cell lines).

Tissue can be isolated from live animals without killing the animal, depending on the target cells. The tissue will be transferred to basal media containing antibiotics, and cells will outgrow the tissue. Another option is mechanical or enzymatic cell dissociation.

##### 2.5.2.2 **Note:***Cryopreservation and thawing*

Cryopreservation provides long-term preservation of the cell lines to ensure that the facility has access to the cell lines if the system fails. Cryopreserved cells at −80 °C lose their viability within months; thus, long-term storage below the glass transmission point of water (−136°) is required. The principle of successful cryopreservation and cell recovery is slow freezing and quick thawing. Freezing should be performed at a rate of −1 to −3 °C per minute, and thawing should be conducted rapidly in a water bath at 98 °F/37 °C for 3–5 min. Successful cryopreservation and thawing can be achieved by:

● Using a healthy culture with more than 90 % viability and no sign of microbial contamination.
● Culture should be in the lag phase of growth (cultures below the maximum cell density should be used to achieve this, and the culture medium could be changed 24 h before freezing).
● High concentrations of serum and protein should be used, but since the goal is to develop a serum-free platform, serum substitutes may be used.

Table 4 presents some of the cryoprotective agents and FDA regulations and codes associated with them.

**Table 4:** Cryoprotective agents and the US FDA regulations and codes.

Chemical	Food grade	FDA Rule	FDA Code
Ethylene glycol	may be safely used in food	The additive is an additional polymer of ethylene oxide and water with a mean molecular weight of 200 to 9,500. It contains no more than 0.2 percent total by weight of ethylene and diethylene glycols when tested by the analytical methods prescribed in the next paragraph. Analytical method. (1) The analytical method prescribed in the National Formulary XV (1980), page 1244, for polyethylene glycol 400 shall determine the total ethylene and diethylene glycol content of polyethylene glycols having mean molecular weights of 450 or higher.	21CFR172.820
Propylene glycol	GRAS	There is no evidence in the available information on propylene glycol and propylene glycol monostearate that demonstrates or suggests reason to suspect a hazard to the public when they are used at levels that are now current or that might reasonably be expected in the future.	21CFR 184.1666
Dimethyl sulfoxide (DMSO)	may be safely used in food	The total dimethyl sulfoxide content is not more than 2 parts per million	21CFR172.859
Glycerol	GRAS		21CFR182.1320
Formamide	Not food grade	Substances permitted for use in adhesives	21CFR175.105
Methanol	Not food grade	Indirect food additive	21CFR176.210
Butanediol	It may be used only if the substance meets the following specifications	1,3-Butylene glycol content: Not less than 99 percent. Specific gravity at 20/20 °C: 1.004 to 1.006. Distillation range: 200–215 °C. It is used in the minimum amount required to perform its intended effect. It is used as a solvent for natural and synthetic flavoring substances except where standards of identity issued under section 401 of the act preclude such use.	21CFR173.220

##### 2.5.2.3 **Note**: *Dedicated liquid nitrogen area*

Using liquid nitrogen is critical for cell culture facilities but the risks associated with liquid nitrogen should be considered. The most important safety concern associated with liquid nitrogen is asphyxiation. Liquid nitrogen refrigerators should be placed in properly ventilated areas, oxygen alarms should be set to 18 % oxygen (v/v), and staff should receive proper training and work in pairs when handling liquid nitrogen. Also, working with liquid nitrogen outside of working hours should be prohibited.

#### 2.5.3 Receiving shelf-stable ingredients

Bag-in-box dried protein, peptides, vitamins, glucose, and salts are received from our sole source provider that offers products only from suppliers complying with the FSMA for plant-based products and certificate of analysis and health for pharmaceutical-based products. A certificate of analysis for aflatoxin and microbial count tests was received.

#### 2.5.4 Receiving refrigerated ingredients

Some of the media ingredients are received in liquid form and require storage in refrigerated conditions (38 F°/4 °C).

#### 2.5.5 Receiving frozen ingredients

Frozen ingredients will be stored at the correct temperature on arrival.

**Note**: The date of ingredient production, date of expiration, lot number, and chemical composition will be checked to avoid receipt of unanticipated allergens. An annual audit of ingredient manufacturers will be conducted as part of the approved supplier program. No medication or antibiotics will be added to the ingredients. All of the media ingredients are food-grade and will be prepared in the facility according to the Good Manufacturing Practices (GMPs). To avoid cross-contamination, ingredients with allergenic components are stored together and labeled as such. Ingredients containing plant and animal-based compounds that might be allergenic are segregated and stored in a separate storage area. Pest control inspections are conducted daily in the ingredient storage room by a trained employee, and pest control is contracted to a third party that visits the facility on a monthly basis. Storage temperatures are continuously monitored, and any deviation from the set control point is recorded with appropriate corrective actions taken.

#### 2.5.6 Receiving plant-based scaffold

Plant-based scaffold will be received from a supplier and stored at room temperature. A certificate of analysis and health will be provided by the supplier, and if any allergen proteins such as soy fibers or wheat have been used, they will be declared by the suppliers and on the final product label.

### 2.6 Process description – preparatory steps

#### 2.6.1 Water and water treatment

The facility has access to both well and city water. UV will be used to treat the water before entering the facility. The water samples will be tested weekly for total coliforms (<4 CFU/ml), and the presence/absence of *Escherichia coli*. UV light intensity is checked weekly to monitor the remaining bulb life following the manufacturer's recommendations. The UV system surface is monitored daily and fouling is removed as needed. The water for media preparation will be filtered by reverse osmosis and resin cartridge purification (final resistance of 16–18 MΩ) before use.

**Note:** If the facility is using public water, it is not required to provide testing results according to US regulations.

#### 2.6.2 Media preparation

Depending on the cells, growth stage, purposes (proliferation/differentiation), the color of the final products, etc., different media formulations will be prepared using the available ingredients. For convenience and to reduce the risk of cross-contamination, media will be prepared as 10X liquid media and then diluted as needed. After media preparation, the pH will be adjusted, followed by filtration and High-Temperature Short Time (HTST-161 °F/73 °C for 15 s, followed by rapid cooling to 8 °F/4 °C) steps before use to inactivate any possible contaminants. Media will be prepared fresh and stored at 38 °F/4 °C for not more than 4 weeks after adding glutamine in sterile air and light-proof jars. The media will be warmed up to 82 °F/28 °C (or the correct temperature for the specific cell line) before adding to the fermenters.

#### 2.6.3 Initial cell culturing

Cryopreserved stem cells will be thawed rapidly in a water bath at 37 °C for 3–5 min and transferred to T-25 and T-5 flasks for culture in basal media such as L15 (galactose, glucose, phenol red, HEPES, l-glutamine, sodium bicarbonate, sodium pyruvate), Dulbecco's Modified Eagle Medium (DMEM) (glucose, calcium chloride, sodium chloride, sodium bicarbonate, monosodium phosphate, magnesium sulfate, amino acids). Cells will be examined for mycoplasma and other microbial contamination during this stage. Routine contamination monitoring will be conducted daily during cell culture for 5 days at 28 °C.

### 2.7 Process description - aseptic cell culture

#### 2.7.1 Personal hygiene

Cell culture personnel must wash their hands on entry to the laboratory and wear gowns and surgical gloves. Surgical gloves must be sanitized with 70 % (v/v) sterile isopropanol. A head cap, face mask, beard cover, and shoe covers should be worn at all times.

The personnel working in the cultured meat facility must be qualified/trained by obtaining the proper education, training, and experience to perform and supervise the manufacture of cultured meat. A written document should establish all the responsibilities of the personnel engaged in cultured meat manufacturing. It is recommended that personnel are frequently trained by qualified individuals/instructors and ensure that employees have adequate knowledge of their particular operations. Records must be maintained for all the employees' training, and they must be periodically assessed.

If any person/employee (by medical examination or supervisory observation) shows or appears to be sick or ill, have an open lesion (including boils, infected wounds, sores, or other characteristics that could be a source of microbial contamination and cause cross-contamination to food or food contact-surfaces, packaging materials) must be excluded from the processing/operation. Then, return to work only if the open lesion is appropriately covered to avoid cross-contamination. If the employee is excluded from any operation, a qualified medical entity or person should verify when this person could go back to their position without compromising the safety or quality of the cultured meat product.

All people in direct contact with cultured meat, food-contact surfaces, and food-packaging materials must comply with hygienic practices while working to protect the cultured meat food against allergen cross-contact and cross-contamination of food. The procedures to maintain cleanliness include:

(1) Personnel must use and wear clean clothing appropriate to the operation to protect the food, food-contact surfaces, or food-packaging materials from allergen cross-contact and cross-contamination. In addition, clothes must be changed when appropriate or needed.
(2) Ensuring adequate personal cleanliness.
(3) Personnel must wash hands carefully (and sanitize when necessary to protect against microbial contamination). The hand-washing procedure must be done before starting to work, after each absence from the workstation, and at any other time when the hands may have become contaminated.
(4) Personnel must remove all unsecured jewelry and other items that could fall into food, equipment, or containers; employees must also remove hand jewelry that cannot be effectively sanitized during periods in which food is manipulated by hand. If the jewelry cannot be removed, it must be covered using a material that ensures the jewelry will be maintained in an intact, clean, and sanitary condition and will effectively protect food, food-contact surfaces, or food-packaging materials against contamination by these items.
(5) If gloves are used when handling the food, gloves must be maintained intact, clean, and in good sanitary conditions.
(6) The personnel must wear hairnets all the time during the production of CM products. And ensure that any types of hair restraints (hair nets, beard covers, or other protective hair) are appropriately worn.
(7) Provide areas for personnel to store clothing or other personal belongings that are not close to where food is exposed or where equipment or utensils are washed. Personal belongings must be restricted to designated locker areas.
(8) Activities such as eating food, chewing gum, drinking beverages, or using tobacco must be confined to designated areas.
(9) Ensure that all precautions are in place to avoid allergen and microorganism cross-contamination.

#### 2.7.2 Microbiology safety cabinet

The airflow in biosafety cabinets does not sterilize the environment but keeps it clean. All the required materials and supplies should be placed in the cabinet before use to restrict the number of times the operator removes their arms or hands from the cabinet. All the items that enter the cabinet should be disinfected with 70 % alcohol solution, and the biosafety cabinets will be located in a Biosafety Level 2 laboratory.

#### 2.7.3 Pipetting

Different-sized sterile disposable plastic pipettes are available for cell culture. Any error in pipetting may introduce microbial contamination, so the following protocols should be used to reduce the risk of contamination:

● Use the automatic pipette aids for a specific cabinet and disinfect the pipette aid regularly.
● Use plugged pipettes when transferring the medium.
● Aerosols can spread contaminations; thus, do not create bubbles in the medium to avoid generating aerosols.
● Clean spills immediately with 70 % (v/v) sterile isopropanol.

#### 2.7.4 Quality control of cells

This section details the quality parameters used for cells.

#### 2.7.5 In vitro pluripotency potential of cells

Pluripotency of cells *in vitro* must exhibit several characteristics such as stable growth during long-term culture, small size, sparse cytoplasm, and large nuclei, normal karyotype, high alkaline phosphatase activity, the ability to form tightly compacted colonies, and the ability to grow undifferentiated. Further cell characterization such as transfection efficiency, proliferation efficiency, cryopreservation, karyotype, and gene expression analysis could be performed using the standard protocols for fish cell lines and meat cell lines.

#### 2.7.6 Cell performance

The most common cell performance parameters are cell viability and cell count but other parameters such as enzymatic activity, genetic markers, cell wall integrity, cell morphology, and karyotype could be used to determine the cell quality.

#### 2.7.7 Contaminant free

Routine sampling should be performed during cryopreservation, cell line receiving from the suppliers, cell culture in flasks, scale-up, differentiation, and scaffold perfusion to determine bacterial and mycoplasma contamination.

#### 2.7.8 Authentication of cell lines

All cell lines should be checked on arrival at the facility. Also, several cell lines may be available for different products in a facility, so the cells need to be checked after thawing.

**Note:** In addition to conventional bacterial culture, molecular methods such as PCR (Roche assays) could potentially be used, as well as testing for specific prevalent stains including *Mycoplasma arginini*, *M. pneumoniae*, *M. genitalium, M. fermentans*, *M. hominis*, *M. hyorhinis*, *M. orale*, and *Acholeplasma laidlawii* [21].

**Note**: Some less common bacterial contaminants in cell cultures, such as Cutibacterium acnes from human skin, are slow-growing and hard to detect in typical monitoring. If contamination is suspected but not detected through routine monitoring, other types of testing may be required.

### 2.8 Process description – cell production and harvesting

#### 2.8.1 Cell proliferation and differentiation

Cells are transferred from the media to fermenters with species-specific growing media; we assumed the general media composition for Atlantic salmon (see Table 1). Fermenters are monitored using continuous real-time temperature, pH, glucose, lactate, ammonia, and cell density measurements, with daily tests for mycoplasma contamination and weekly tests for bacteria such as *E. coli, Salmonella* spp., and *Listeria monocytogenes*. During cell handling, workers are required to wash and sanitize their hands, as well as wear the necessary personal protective equipment, including lab coats, hairnets, face masks, gloves, and sanitized boots. Cells need to be differentiated into muscle and fat cells in cell proliferation tanks. In this step, food-grade chemicals are applied to induce differentiation, and no other non-food-grade chemicals, including dexamethasone, should be added. Cell differentiation is monitored on a daily basis until the desired differentiation is reached.

#### 2.8.2 Biofilters for spent media recirculation

Biofilters convert ammonia to nitrite and nitrite to nitrate. Since nitrate is 400 times less toxic to fish cells than ammonia, it can be utilized by plants as a nitrogen source. *Nitrosomonas* spp. convert ammonia to nitrite and *Nitrobacter* spp. convert nitrite to nitrate, and 4.5 g of oxygen is required to convert 1 g of ammonia to nitrate. Bacterial survival in the biofilters depends upon maintaining the correct dissolved oxygen level (at least two mg/L), alkalinity (50–100 mg/L CaCO_3_), pH (above 7), and temperature (around 68 °F/20 °C) [22]. A U-tube or a blower provides oxygen to the biofilter system, and the water recovered from the biofilters is passed through a UV unit or treated with ozone. After UV treatment, spent media is mixed with fresh media (10X) (9:1 v/v) and filtered to remove any possible contamination.

#### 2.8.3 Cell harvesting

After differentiation, fish cells are harvested directly from the tanks with a filter by workers in sanitized uniforms wearing clean gloves and personal protective equipment.

#### 2.8.4 Washing

Fish cells are washed with clean, cold water and then transferred to either cell transportation packages or used in 3D printing of the whole tissue to prepare salmon muscle portions. The processing room has positive pressure to reduce the risk of environmental microbial contamination, and the temperature is < 50 °F/10 °C. Sanitary ice is on hand if ambient temperatures are too high to keep the fish cells cold.

### 2.9 Process description – food processing

#### 2.9.1 Fish cell processing and shipping

Packages are affixed with a unique number seal that is recorded and sent in advance to the customer for verification upon arrival as part of traceability and food defense programs. The temperature during shipment is controlled to maintain fish cell quality at 38 °F (4 °C) or frozen. The temperature is monitored using data loggers.

#### 2.9.2 Bioprinting fish tissue

Cells will be used to develop the bio-ink for 3D printing of the whole fish tissue. Cells are mixed with bio-ink containing food-grade hydrogels, collagen, xanthan, curdlan, alginate, wheat, soy, pea, and algae oil [23]. Three nozzle bioprinters will print a whole fillet at 38 °F/4 °C and the nozzles are changed between each production batch and washed and sanitized at the end of the shift. The formulation holder, whole-cut printing stand, and bio-ink preparation vessels are washed and sanitized at the end of each production cycle.

#### 2.9.3 Cell infusion to a plant-based scaffold

After preparing the cell biomass, cells can be assembled on the surface of a plant-based scaffold prepared by decellularization or fabrication. Different types of scaffolds, including porous, fiber, and hydrogel structures, could be used and if allergens such as soy or wheat are included, this should be mentioned on the label. Synthetic polymers, as well as gelatin, collagen, cellulose, alginate, gums, and chitosan, could be used for scaffold development if FDA-approved. Decellularization methods require chemical applications to remove the cells from the plant tissues, and these chemicals, including sodium dodecyl sulfate, are generally recognized as safe and food-grade. Cell adhesion to the scaffold could be performed by using food-grade polymers such as alginate, gelatin, and synthetic polymers, including Polycaprolactone (PCL) and Poly(lactic-co-glycolic) acid (PLGA). Adhesion motifs such as arginyl-glycyl-aspartic acid (RGD) could also be used alone or in combination with cellulose. Peptides significantly improve the adhesive properties of scaffolds such as food-grade proline-histidine-serine-arginine-asparagine sequences (PHSRN), and glycine-phenylalanine-hydroxyproline-glycine-glutamic acid-arginine (GFOGER). Laminin-derived domains are another promising strategy for improving the cell-adhesive properties of certain materials. Cell infusion is conducted at 38 °F/4 °C.

#### 2.9.4 Weighing, packing, and labeling

Cells or final fish portions will be weighed, packaged, and labeled properly. Boxes are labeled with lot/batch number, fish species, production location, address, contact information, and production date, and then stored at less than 38 °F/4 °C or shipped in refrigerated containers. Temperature is monitored during shipment with a thermometer, a data logger, or the presence of adequate ice. All the packages will be labeled as an allergen (containing fish, wheat, soy, and egg albumin). If reduced oxygen packaging (ROP) is used, more instructions will be provided for users regarding the defrosting of the package at a cold temperature and opening the box before thawing to control *Clostridium botulinum* germination. Another method for controlling the growth of *C. botulinum* is using packaging materials with an FDA-approved OTR, which should be more than 10,000 cc/m^2^/24 h at 24 °C.

#### 2.9.5 Cold storage

The products are stored in a cold room (34–38 °F/1.5–4 °C) until transferred to the market, which occurs within 2 days.

#### 2.9.6 Shipping

Products are shipped refrigerated to the market by a carrier and first in first out (FIFO) rules apply to the distribution. The vehicle for shipment is evaluated to meet the requirements for sanitary transport (21 CFR Parts 1 & 11).

**Note:** According to 21 CFR Part 123 (Seafood HACCP) and 21 CFR Part 117, the bioprinter will be handled by workers who have been trained in good manufacturing practices and sanitation and hygiene practices.

**Note:** The facility has restrooms in the factory and each processing room that are equipped with handwashing sinks, sanitizers, and disposable paper towels, meeting the requirements of 21 CFR Part 117 & 123.

## 3 Hazard analysis

### 3.1 Fish cell production

In addition to Table 5, it is crucial to address the significance of protozoan parasites in terms of food safety. Various protozoan parasites, including *Toxoplasma gondii*, *Trypanosoma cruzi*, *Leishmania* spp., *Cryptosporidium parvum*, and *Plasmodium* spp., can emerge during primary cell line development [24,25]. This emergence poses challenges to established cell lines and elevates the food safety risks [7]. Identification of protozoans relies on conventional microscopic techniques during cell line development, focusing on their shape, size, and mobility. However, certain protozoans, like *Ichthyobodo*, might evade detection when using 100X microscopes [26], potentially persisting in cell cultures. This persistence can lead to delays in cell adhesion and growth while also heightening food safety concerns. Different strategies could be used to address the concerns associate with protozoans including preventive control measures. These preventive controls may include:

- Supplying samples from fish sourced from accredited providers with health certificates.
- Sampling: Protozoa may adhere to the skin, fins, and gills, potentially contaminating tissue during the cell line development process. Implementing appropriate sampling techniques, such as surgically removing muscle tissue instead of punching, would significantly decrease the risk of cross-contamination.
- Apply FDA-approved chemicals (Formalin-F®, Paracide-F®, Parasite-S®, Potassium permanganate, Copper sulfate, sodium chloride, Calcium oxide, Acetic acid) [7,26] for disinfecting fish tissues prior to sampling for cell line development.

**Table 5:** Hazard analysis example form for fish cell biomass.

Food Safety Plan – Hazard Analysis Cell Mass Production				Cell Factory Atlantic Salmon Cell Production				
Cell-Cultured Salmon Company								
Approved by: Signature:				Version 2: Sept. 9, 2022				
(1) Ingredient/processing step	(2) Identify potential food safety hazards introduced, controlled, or enhanced		(3) Do any potential food safety hazards require preventive control?		(4) Justify your decision for column 3	(5)What preventive control measure(s) can be applied to significantly minimize or prevent the food safety hazard? Processes include CCPs, allergens, sanitation, supply chain, other preventive controls	(6) Is preventive control applied at this step?	
			Yes	No			Yes	No
Receiving packaging	B	None						
	C	None						
	P	None						
	A	None						
	E	None						
	I	None						
Receiving shelf stable ingredients	B	Biological hazard: Vegetative pathogens	X		Dried ingredients such as soy protein may have a history of potential contamination with pathogens	Supply chain control: Microbial contamination is controlled by the supplier through thermal processing	X	
		Spore Forming pathogens such as *Clostridium botulinum*		X	*C. botulinum* will not grow or produce toxin on dried products			
	C	Chemical hazards: Aflatoxin	X		Soy and fish proteins have a history of aflatoxin contamination	Supply chain control: Supplier verification, and certificate of analysis	X	
		Pesticides		X	Ingredients are US-based products, and FDA data rarely show pesticide residues			
	P	None						
	A	Allergens: such as soy, wheat, fish peptides	X		Soy, wheat and fish are allergens	Allergen control: Cross contact prevention, and labeling in next steps		X
	E	Economic fraud	X		History of fraud and mislabeling	Supply chain control: Supplier verification, certificate of analysis and third party audit	X	
	I	Intentional contamination	X		History of added chemicals	Supply chain control: Supplier verification, certificate of analysis and third party audit	X	
Receiving refrigerated ingredients	B	Biological hazard: Mycoplasma	X		History of mycoplasma contamination	Supply chain control: Supplier verification and certificate of analysis	X	
		Spore Forming pathogens such as *Clostridium botulinum*		X	*C. botulinum* will not grow or produce toxin on refrigerated products			
	C	Aflatoxin		X				
		Pesticides		X				
	P	None						
	A	Chemical hazard: Allergens such as albumin from egg	X		Albumin from egg	Allergen control: Cross contact prevention, and labeling in next steps		X
	E	Economic fraud	X		History of fraud and mislabeling	Supply chain control: Supplier verification, certificate of analysis and third party audit	X	
	I	Intentional contamination	X		History of added chemicals	Supply chain control: Supplier verification, certificate of analysis and third party audit	X	
Receiving frozen ingredients	B	Biological hazard: Mycoplasma	X		History of mycoplasma contamination	Supply chain control: Supplier verification and certificate of analysis	X	
		Spore Forming pathogens such as *Clostridium botulinum*		X	*C. botulinum* will not grow or produce toxin on refrigerated products			
	C	Aflatoxin		X				
		Pesticides		X				
	P	None						
	A	Chemical hazard: Allergens such as albumin from egg	X		Albumin from egg	Allergen control: Cross contact prevention, and labeling in next steps		X
	E	Economic fraud	X		History of fraud and mislabeling	Supply chain control: Supplier verification, certificate of analysis and third party audit	X	
	I	Intentional contamination	X		History of added chemicals	Supply chain control: Supplier verification, certificate of analysis and third party audit	X	
Receiving plant-based scaffold	B	Pathogenic bacteria growth		X	Bacteria cannot grow on low moisture food materials			
	C	None						
	P	None						
	A	Allergens such as soy	X		Soy protein is allergen	Allergen control: Cross contact prevention, and labeling in next steps		X
	E	None						
	I	None						
Media preparation	B	Biological hazard: pathogenic bacteria such as *Listeria monocytogenes, E. coli*	X		History of pathogenic bacteria contamination	Supply chain control: Supplier verification and certificate of analysis Post-preparation quality control HTST temperature and time control	X	
		Mycoplasma	X		History of mycoplasma	Supply chain control: Supplier verification and certificate of analysis Post-preparation quality control HTST temperature and time control	X	
		Spore Forming pathogens such as *Clostridium botulinum*		X	*C. botulinum* will not grow or produce toxin on refrigerated and frozen products			
	C	Aflatoxin		X				
		Pesticides		X				
	P	None						
	A	Allergens such as albumin from egg, soy, wheat, fish peptides	X		Soy, wheat, egg, and fish are allergens	Allergen control: Cross contact prevention, and labeling in next steps		X
	E	Economic fraud		X				
	I	Intentional contamination	X		History of added chemicals	Supply chain control: Supplier verification, certificate of analysis and third party audit	X	
Receiving Cells from Cell Bank-Cryopreserved Cells	B	Biological hazard: Microbial contamination Cell line Contamination with other Cell lines Mycoplasma, *L. monocytogenes*	X		History of contamination	Supply chain control, approved third-party supplier audit if a third party provides the cell lines Microbial testing of fish as part of health certificate sent with each lot of fish cells Every new cell line is cultivated in a quarantined incubator and cells are verified that they are pathogen-free before use in production. Any contaminated cells are destroyed	X	
	C	Chemical hazard: Antibiotics, Cryoprotectants	X		Unapproved antibiotics; Food grade cryoprotectants to be used: inulin, sorbitol, and dimethyl sulfoxide	Supply chain control, approved supplier, third-party audit if a third party provides the cell lines	X	
	P	Physical hazard: foreign materials		X	Unlikely for cell lines			
	A	Allergen contamination		X	Allergens such as albumin from egg, soy, wheat, fish peptides	Allergen control: Cross contact prevention, and labeling in next steps		
	E	Economic fraud: different species	X		Cell lines from other species could pose allergen risk	DNA barcoding and tests and third-party approval	X	
	I	Intentional contamination		X	Low risk because toxicants likely would affect cell survival	Supply chain control program		
Culturing Cells in Fermenter/Reactor Cell Proliferation	B	Biological hazard: contamination Cell line Contamination with another Cell line, such as feeder layer cells Mycoplasma, *A. hydrophila,* *L. monocytogenes,* *Vibrio* spp., *Salmonella* spp., Pathogenic *E. coli*	X		Risk of foodborne outbreak	Chain of custody for culture material Mycoplasma testing on a daily basis Microbial testing every two weeks	X	
	C	Chemical hazard: animal drugs or unapproved chemicals; leachable chemicals from sensors, equipment, piping, surfactant, anti-foaming agents Animal origin growth factors	X		Unapproved Antibiotics Chemicals Unapproved animal origin growth factors	No unapproved Antibiotics Routine checking the system Supply chain control, approved third-party supplier audit if third party provides the media ingredients	X	
	P	Physical hazard: foreign materials		X	Easily removed if present			
	A	Allergen contamination		X	No allergens introduced			
	E	Economic fraud		X	Species substitution	Species identification verified when cell lines were received		
	I	Intentional contamination	X		During the production, any chemicals can be added intentionally	Security and monitoring; Cybersecurity program and policy; Personnel training; Only authorized people have access to the fermenters and production room; Only authorized people have access to the process control room	X	
Cell Differentiation	B	Biological hazard: microbial contamination and growth: Mycoplasma, *A. hydrophila,* *L. monocytogenes,* *Vibrio* spp., *Salmonella* spp., Pathogenic *E. coli* Genotoxicity	X		Risk of a foodborne outbreak not likely since operating under sterile conditions	Supply chain control, approved third-party supplier audit if a third party provides the cell lines Microbial testing of fish as part of health certificate sent with each lot of fish cells DNA testing		X
	C	Chemical hazard: antibiotics Non-food grade differentiation agents	X		Possibility of harmful residues in cells	Dexamethasone is not used as a differentiation agent – check for the presence of this compound	X	
	P	Physical hazard: debris		X	Easily removed if present			
	A	Allergen contamination		X	No allergens introduced			
	E	Economic fraud		X				
	I	Intentional contamination	X		During the production, chemical contaminants could be can be added intentionally	Security and monitoring; Cybersecurity program and policy; Personnel training; Only authorized people have access to the fermenters and production room; Only authorized people to have access to the process control room	X	
Spent Media Recirculating System-Biofilter	B	Biological hazard: microbial contamination	X		Microbial contamination	GMPs control spurious microbial growth	X	
	C	Chemical hazard: Contamination	X		Ammonia levels high	Collect samples from the reactor to measure ammonia, nitrite, and nitrate as part of GMPs Lactate level should be controlled A cell health factor but not a food safety risk	X	
	P	Physical hazard: foreign materials		X	Biofilter should not be clogged	Regular checking of the biofilter to remove any solid materials		
	A	Allergen contamination		X	No allergens introduced			
	E	Economic fraud		X	Low risk for a food safety hazard			
	I	Intentional adulteration		X	Enclosed and difficult to contaminate			
Spent Media Recirculating System-UV	B	Biological hazard: microbial contamination and growth	X		No media sanitation/poor sanitation technique	After removing chemicals in a bioreactor, media will be sanitized by UV, and samples will be collected for sanitation verification	X	
	C	Chemical hazard: Lactate		X	No chemicals used at this step Lactate might be concentrated	GMPs		
	P	Physical hazard: foreign materials		X	Unlikely	Settled out or removed by filters		
	A	Allergen contamination		X	No allergens introduced	GMPs control the water distribution system No cross-connections		
	E	Economic fraud		X	Low-quality system functionality easily detected			
	I	None						
Spent Media Recirculating System-Filtration	B	Biological hazard: microbial contamination and growth	X		No media sanitation/poor sanitation technique	After removing chemicals in a bioreactor, media will be sanitized by UV and will be filtered. Samples will be collected for sanitation verification	X	
	C	Chemical hazard		X	No chemicals used at this step Lactate might be concentrated			
	P	Physical hazard: foreign materials		X	Unlikely	Settled out or removed by filters		
	A	Allergen contamination		X	No allergens introduced	GMPs control the water distribution system No cross-connections		
	E	Economic fraud		X	Low-quality system functionality easily detected			
	I	None						
Cell harvesting	B	Biological hazard: microbial contamination	X		History of cross contamination	Routine water testing program in place		X
	C	None						
	P	None						
	A	Allergen contamination	X		Fish products are allergens			X
	E	Economic fraud		X				X
	I	Intentional contamination: access to cells		X	Cells are held in a secured area	Control access to approved individuals only		X
Washing	B	Biological hazard: microbial contamination	X		Poor water sanitation, and cross contamination during the washing step may introduce bacteria	Routine water testing program in place, routine UV and filtration water treatment GMPs training for food handlers	X	
	C	None						
	P	None						
	A	None						
	E	None						
	I	None						
3D Bioprinting, cell diffusion to the scaffolds	B	Biological hazard: microbial contamination, cross contamination and growth	X		Due to time and temperature abuse, and cross contamination bacteria may grow	Supply chain verification Temperature control Microbial testing of hydrogels and bio inks Cell diffusion process temperature control	X	
	C	Chemical hazard: contamination	X		Unapproved and non-food grade additives	Supply chain control, approved third-party supplier audit	X	
	P	Physical hazard: foreign material	X		Debris, foreign matter	GMP; physical inspection on receipt		X
	A	Allergen contamination		X		No allergen other than fish		X
	E	Economic fraud		X		GMPs and technical specifications followed		X
	I	Intentional contamination		X	Unapproved materials, and colorants may be added	Authorized personnel have access to the final product room; GMP is followed	X	
Weighing, packing, labeling	B	Vegetative bacteria growth		X	Short process time, pathogens unlikely to grow			
		Spore Forming bacteria *(C. botulinum)* growth	X		Reduced Oxygen Packaging (ROP) creates anerobic environment for C. botulinum to grow if time and temperature abuse occurs	Proper labeling and instruction regarding thawing Using proper packaging materials with FDA approved Oxygen Transmission Rate (OTR)-10,000 cc/m^2^/24 h at 24 °C, or higher	X	
		Product cross contamination	X		Poor equipment cleaning, and employees hygiene	Sanitation control program for zone 1 and 2, and environmental sanitation	X	
	C	None						
	P	None						
	A	Allergens	X		Product containing Aquatic species, egg, soy and wheat are allergens	Allergen control by declaration on label	X	
	E	Economic fraud	X		Mislabeling	Proper labeling program and records keeping	X	
	I	None						
Cold storage	B	Vegetative and spore forming pathogen growth	X		Pathogens can grow due to time and temperature abuse	Process control for product and shipping vehicle temperature with data logger	X	
	C	None						
	P	None						
	A	Allergens		X	Fish, egg, soy, and wheat are allergens	Proper labeling in previous step		
	E	None						
	I	None						
Shipping	B	Vegetative and spore forming pathogen growth	X		Pathogens can grow due to time and temperature abuse	Using data logger for shipping	X	
	C	None						
	P	None						
	A	Allergens		X	Fish, egg, soy, and wheat are allergens	Proper labeling in previous step		
	E	None						
	I	None						

^1^ For each ingredient, such as soy, wheat, and fish peptides, individual sections should be added under dry ingredients.

The FDA has devised control strategies for parasites in seafood, which could also be applied to cell-based seafood products should any parasites persist [7]. Freezing and storing at an ambient temperature of −20 °C or below for 7 days is an effective method for controlling parasites in seafood products when consumed raw.

### 3.2 Temperature monitoring preventive controls

A temperature monitoring table will be employed to oversee temperature levels, identify instances of temperature irregularities, establish preemptive measures, enact corrective actions, and document and validate data. This tool will enable producers and inspectors to vigilantly track temperature fluctuations and preempt potential food safety concerns (Table 6).

**Table 6:** Temperature monitoring preventive controls.

Food Safety Plan – Temperature monitoring Preventive Controls						Cultivated Atlantic salmon (*Salmo salar*)				
Approved by: Signature: Processing Manager						Version 2: Sept. 9, 2022				
(1) Temperature control	(2) Hazard	(3) Criterion	Monitoring					(8) Corrective action	(9) Records	(10) Verification
			(4) What	(5) How	(6) Frequency		(7) Who			
Fish	Temperature abuse	The product must be kept under 38 °F/4 °C	Temperature monitoring	Trained employee checks storage room, processing room, and shipping data loggers	Beginning and end of each shift and on lot changes		Quality assurance lead	There are always three sensors for each section, and data will be monitored real time, if the temperature changes, system will send alerts. Sensors will be checked, and the area temperature will be monitored by hand-held sensor Root cause analysis conducted to determine why failure occurred, and issue is corrected	Processing log	Sensors will be checked Refrigeration systems will be evaluated by third party

### 3.3 Allergen preventive controls

Allergen preventive control is a valuable tool for identifying allergens, developing preventive measures where feasible, ensuring proper labeling strategies, and maintaining thorough records for verification purposes (Table 7, Table 8).

**Table 7:** Food safety plan: Allergen preventive controls for fish products.

Food Safety Plan – Allergen Preventive Controls					Cultivated Atlantic salmon (*Salmo salar*)				
Approved by: Signature: Processing Manager					Version 2: Sept. 9, 2022				
(1) Allergen control	(2) Hazard	(3) Criterion	Monitoring				(8) Corrective action	(9) Records	(10) Verification
			(4) What	(5) How	(6) Frequency	(7) Who			
Fish	Undeclared allergen	The product must contain an allergen declaration: “INGREDIENTS: Fish: Atlantic salmon”	Presence or absence of allergen declaration	Trained employee (packaging lead) checks product labeling at the beginning and end of each shift and on lot changes Trained employee records observations on packaging log	Beginning and end of each shift and on lot changes	Packaging lead	In the event that the allergen declaration is not on the label, the packaging lead will notify the supervisor and place the affected product on hold until the last good check The product is then properly labeled Root cause analysis was conducted to determine why the failure occurred, and the issue is corrected	Packaging log	Label review Labeling records are reviewed and verified by QC

**Table 8:** Cell-based fish products labeling.

Products		Allergen Statement							
Fish cell biomass		INGREDIENTS: Atlantic salmon (*Salmo salar*)							
Product name	Production line	Intentional Allergens							
		Egg	Milk	Soy	Wheat	Tree nut	Peanut	Fish	Shellfish
Atlantic Salmon	All facility equipment	N/A	N/A	N/A	N/A	N/A	N/A	Declared	N/A

### 3.4 Sanitation preventive controls

#### 3.4.1 Facility sanitation monitoring master list

Table 9 represents the facility sanitation monitoring master list which can be used as a general document for monitoring the sanitation program.

**Table 9:** Sanitation preventive control: Facility sanitation monitoring master list.

Equipment	Process monitor	SOP/form	Monitoring frequency	Action limit	Correction	Records/location	Person responsible	Monitoring record review	Person responsible
Product contact surfaces	Visual inspection	Daily pre-op inspection log	Daily	Visible soil	Reclean and resanitize until visible soil is removed	QC office	Department manager	Daily pre-op inspection log	QC manager
Non Product contact surfaces	Visual inspection	Master sanitation schedule	As determined on the schedule	As determined on the schedule	Reclean and resanitize until visually clean	QC office	Production employees	Master sanitation schedule	QC or department manager

Each cultured meat facility must provide adequate sanitary services and accommodations, including:

#### 3.4.2 Facility sanitation verification activities

Table 10 provides a master list for verification of sanitation activities. Any sanitation must be verified through proper methods.

**Table 10:** Sanitation preventive controls: Facility sanitation verification activities.

Equipment/record	Verification activity	SOP/form	Frequency	Action limit	Person responsible	Corrective action	Implement	Person responsible	Records/location
Product contact surfaces	Adenosine triphosphate (ATP) swabbing	SSOP ATP swabs	Daily	0-10RLU: Pass 11-30 RLU: Caution 31 RLU <: Fail It varies; check the kit manufacturer manuals	Production employee	Reclean and resanitize until ATP results meet the action limit	N/A	QC manager	QC office

#### 3.4.3 Facility sanitation implementation/effectiveness

Facility sanitation implementation and effectiveness is another valuable tool for the industry to control pathogenic bacteria control. Calibration and calibration frequencies might be different depending on the tool, and facility strategies (Table 11).

**Table 11:** Sanitation preventive controls: Facility sanitation implementation/effectiveness.

Test	Equipment/reagent	Calibration	Calibration frequency	SOP #	Person responsible	Records/location	Monitoring record review	Person responsible
Environmental swabbing (*Listeria* spp.)	Sponge stick swab	Controls are run by the outside laboratory for the methods used to test the sponge	Daily	Provided by laboratory	QA manager	QC office and outside laboratory	QC manager	QC manager
ATP swabbing and testing	Nova LUM II	Positive and negative controls	Negative control = monthly Positive control = monthly	ATP swabbing and testing	Quality department associate or designee	Quality department	ATP controls: performance checks	Quality department associate or designee

## 4 General assessment information

### 4.1 Food protection plan

All employees should be trained according to the 21 CFR Part 11, 117, 121, and 123 requirements. One trained person per shift should serve as a PCQI, and individuals who have had seafood HACCP training should become familiar with the new requirements of FSMA. These individual(s) will be required to prepare the food safety plan, develop the hazard analysis, validate the preventive controls, review food safety and food protection records, and conduct a reanalysis of the food safety plan. A qualified individual will also understand the importance of sanitation and biosecurity and can implement an effective plan to reduce the risks of human, animal, and plant diseases. There will be at least one qualified individual on-site at all times who has an understanding of the intentional adulteration rule and how that is to be implemented at our facility. All employees should have some aspect of food safety training which will be documented with records retained in personnel or contractor files. Training for all employees is updated as hazard analyses change or at least twice per year. The following table provides an assessment rubric showing when a food protection plan is satisfactory and when it is not.

### 4.2 Cybersecurity

More than 20 % of the domestic bio-economy in the US is related to food and agriculture, with the data science market around $20 B. The cell-cultured meat industry is relatively new and could be more vulnerable since cyber-attacks often target organizations with less than 100 employees. The cell-cultured meat industry is a cell factory depending on real-time, point-of-use, alarms, cyber-physical systems, the Internet of Things (IoT), and smart decision tools. Without proper cybersecurity systems, hackers could jeopardize the system's security, impede the shipping of ingredients, and change the sensor sensitivity ranges causing food security and food safety concerns. Thus, cybersecurity processes are essential for cell factories and the cell-cultured meat industry to maintain production and protect food products and public health. There are three major types of cybersecurity targeting the food industry including Web Skimming, Ransomware, and ICS/SCADA Malware that could be prevented by:

● A comprehensive audit and identifying any gaps in the system.
● Auditing the third-party partners and suppliers.
● Develop a cloud system.
● Use multilayers of data security.
● Develop a data inventory, identify highly sensitive data, and control access to data.
● Have a robust data backup plan.
● Develop standards and a roadmap.
● Develop a cybersecurity team and communicate with them.
● Schedule regular mock penetrating.
● Have an emergency standard protocol for data loss.
● Develop a detection program and detection/breach team (could be a part of the cybersecurity team).
● Follow industry-specific compliance, including FDA and USDA.
● Update antivirus software and systems regularly.
● Do not use unknown drives.
● Inspect each new device's security capabilities when implementing IoT.

### 4.3 Complaints and recalls

Complaints about safety and quality should be recorded and investigated according to a written procedure whether received orally or in writing.

The recommendations for complaint records include:

● Name and address of the complainant
● Name and phone number of the person submitting the complaint
● Complaint nature (including batch and name of the product)
● Date complaint is received
● The action was initially taken (including the dates and identity of the person taking action)
● Any follow-up action taken
● Response provided to the originator of complaint (including date response sent)
● The final decision on the CM batch or lot
● Retaining records of complaints to evaluate tendencies, product-related incidences, and severity will help determine corrective actions
● It is recommended to have a written procedure in place that describes the conditions for considering a CM product recall
● The recall procedure should have the following information: the person in charge to evaluate the situation, how to initiate a recall, who and how the recall will be informed, and what to do with the recalled product

When a recall is considered a potentially life-threatening case, this should be informed to local, national, and international authorities based on the product distribution.

## 5 Conclusion

The cell-based food industries are relatively new, thus, there is a lack of clarity regarding food safety, regulations, food law, and policy. In this review-guidance article, cell-based food safety concerns were addressed and two food safety plans were developed based on two different possible products, including cell biomass to be used for incorporating into other foods such as burgers, patties, or as a flavor enhancer in specific foods; and fish portions (small fish fillet). In the process description, best cell culture practices, cell bank, cryopreservation, and chemicals that might be used for cryopreservation were also discussed. One of the major differences between the food safety plan for cell-based fish and conventional fish is the starting point. In the conventional seafood industry, fish are received whole, alive or dead from fishermen, or aquaculture farms, along with a health certificate. However, in the cell-based food industry, cells are the starting point, and they are received either from a vendor cell bank or the facility cell bank along with several different certificates, including species authentication and DNA barcoding verification. In the conventional seafood industry, most hazards are associated with the processing, while for the cell-based food industry, cell proliferation and differentiation in bioreactors or fermenters are the main part of the processing line. Labeling of the final products depends on the type of packaging and the allergens disclosure. For example, regardless of the industry, if ROP is used for packaging, more instruction should be provided for consumers regarding thawing. This is the first food safety plan for cell-based food that provides in-depth information for researchers, industry, and regulatory agencies regarding cell-based food safety plans.

## Data availability

No data was generated or used for this article.

## CRediT authorship contribution statement

**Reza Ovissipour:** Writing – review & editing, Writing – original draft, Visualization, Validation, Supervision, Resources, Project administration, Methodology, Investigation, Funding acquisition, Formal analysis, Data curation, Conceptualization. **Xu Yang:** Writing – review & editing. **Yadira Tejeda Saldana:** Writing – review & editing. **David L. Kaplan:** Resources, Funding acquisition, Conceptualization. **Nitin Nitin:** Writing – review & editing, Conceptualization. **Alex Shirazi:** Writing – original draft. **Bill Chirdon:** Writing – review & editing. **Wendy White:** Writing – review & editing. **Barbara Rasco:** Writing – review & editing, Writing – original draft, Conceptualization.

## Declaration of competing interest

The authors declare the following financial interests/personal relationships which may be considered as potential competing interests:Reza Ovissipour reports financial support was provided by 10.13039/100000199US Department of Agriculture. If there are other authors, they declare that they have no known competing financial interests or personal relationships that could have appeared to influence the work reported in this paper.
